# Dosimetric effect of internal error correction on pubic bones in image-guided passive scattering proton therapy for prostate cancer

**DOI:** 10.1093/jrr/rraf061

**Published:** 2025-10-06

**Authors:** Kimihiro Takemasa, Takahiro Kato, Sho Sasaki, Yuki Narita, Tomohiro Ikeda, Shuta Ogawa, Sho Oyama, Masao Murakami

**Affiliations:** Department of Radiation Physics and Technology, Southern Tohoku Proton Therapy Center, 7-172 Yatsuyamada, Koriyama, Fukushima, 963-8052, Japan; Department of Radiation Physics and Technology, Southern Tohoku Proton Therapy Center, 7-172 Yatsuyamada, Koriyama, Fukushima, 963-8052, Japan; Department of Radiological Sciences, School of Health Sciences, Fukushima Medical University, 10-6 Sakaemachi, Fukushima, Fukushima, 960-8516, Japan; Department of Radiology, Sapporo Teishinkai Hospital, 3-1, Kita 33-jo Higashi 1-chome, Higashi-ku, Sapporo-shi, Hokkaido, 065-0033, Japan; Department of Radiation Physics and Technology, Southern Tohoku Proton Therapy Center, 7-172 Yatsuyamada, Koriyama, Fukushima, 963-8052, Japan; Department of Radiation Physics and Technology, Southern Tohoku Proton Therapy Center, 7-172 Yatsuyamada, Koriyama, Fukushima, 963-8052, Japan; Department of Radiation Physics and Technology, Southern Tohoku Proton Therapy Center, 7-172 Yatsuyamada, Koriyama, Fukushima, 963-8052, Japan; Department of Radiation Physics and Technology, Southern Tohoku Proton Therapy Center, 7-172 Yatsuyamada, Koriyama, Fukushima, 963-8052, Japan; Department of Radiation Oncology, Southern Tohoku Proton Therapy Center, 7-172 Yatsuyamada, Koriyama, Fukushima, 963-8052, Japan

**Keywords:** prostate cancer, proton therapy, internal error correction, pubic bones

## Abstract

Image-guided passive scattering proton therapy (PSPT) has been widely adopted in Japan and worldwide, with substantial long-term clinical data supporting its efficacy in treating prostate cancer. However, as hypofractionated protocols become increasingly common, the impact of internal anatomical shifts on surrounding organs at risk (OARs) warrants renewed attention. The pubic bones, situated near the prostate, are often exposed to unintended high doses, especially during internal error correction based on fiducial marker alignment. This study retrospectively analyzed 30 patients with localized prostate cancer treated with PSPT using lateral opposed fields. Simulated isocenter shifts were applied anteriorly and inferiorly in 2-mm increments up to 10 mm to assess dose changes to the pubic bones. Dose-volume histogram metrics including V_80%_, V_90%_ and V_95%_ were evaluated. Pubic bones dose increased in both shift directions, with a more pronounced effect for anterior shifts, with a 10-mm anterior shift increasing V_80%_ by 14.2 cc on average—2.4 times greater than inferior shifts. Dose elevation correlated strongly with the anatomical proximity between the clinical target volume and pubic bones (*r* > 0.66, *P* < 0.001). These results suggest that anterior correction in PSPT can cause substantial dose escalation to the pubic bones, potentially increasing the risk of insufficiency fractures. As extreme hypofractionation becomes more common, careful evaluation of pubic bones dose should be incorporated into treatment planning, alongside traditional OARs such as the rectum and bladder. Early replanning should be considered when persistent anterior displacement is observed to maintain patient safety and quality of life.

## INTRODUCTION

External radiation therapy for prostate cancer is widely performed as one of the standard treatments [1]. In recent years, the precision of irradiation methods has advanced, and not only intensity-modulated radiation therapy (IMRT) and stereotactic body radiation therapy, but also proton therapy (PT) has become widespread [2]. The history of PT for prostate cancer is relatively long, and clinical results of long-term follow-up have also been reported [3–5]. Irradiation methods for PT are broadly divided into passive scattering (PS) methods and pencil beam scanning (PBS) methods, with the latter becoming the mainstream in recent years [6]. However, there are still quite a few facilities that use the PS method. Most reports on long-term follow-up of PT have been based on the PS method due to historical reasons, and the actual situation is that PBS is not yet sufficient. In this way, the PS method has already been proven to produce relatively stable results, so it is reliable and is still used at a certain rate. In the PS method, in addition to image-guided radiation therapy (IGRT) using fiducial markers, hydrogel spacer is also widely used [7] and some facilities are actively performing on hypofractionation protocol [2, 8–11]. The trend toward hypofractionation in PT for prostate cancer is expected to accelerate and become routine in the future. However, extreme hypofractionation may require consideration of factors that have not been anticipated thus far, and therefore a more cautious approach will be necessary.

The rectum, bladder, small bowel, large bowel and femoral heads are typical organs at risk (OARs) for prostate cancer in external radiation therapy, but other soft tissues and pelvic bones also need to be considered because of the high-dose prescription. The femoral heads are a representative bone of the OAR in prostate cancer external radiation therapy, and the risk of fracture and osteomyelitis has been discussed [12–15]. Conversely, although the frequency is rare, it has been pointed out that insufficient fractures of the pubic bones can also occur with external radiation therapy [16, 17]. Insufficiency fractures are well known as a late adverse effect of whole pelvic irradiation especially for gynecological diseases [18–22], but are less well understood with external beam radiation therapy for prostate cancer [12–17]. Pelvic insufficiency fractures are predominantly located in weight-bearing areas and have a relation to higher radiation doses with an increasing incidence with age and postmenopausal status [23]. Further, the most frequent localizations were reported in the weight-bearing areas: sacral body/near sacroiliac joint (60–73.6%) and pubic bones (12–13%) [17]. Although there are some reports analyzing the relationship between fracture risk and radiation dose to the femoral heads and sacral bones [18, 24], there are few reports examining pubic bones radiation dose. Kronborg *et al*. reported that there was a borderline significant difference between the fracture and non-fracture groups in the volume of the pubic bones exposed to 40 Gy or more [24]. Rasmusson *et al*. evaluated the pubic bones dose, but they did not mention the details, and there is a complete lack of information on dose constraint of pubic bones [15].

At our institution, parallel opposed lateral fields technique with PS method is performed for prostate cancer [25]. Due to the characteristics of the PS method [26, 27], the pubic bones dose tends to be higher than that of IMRT, so we consciously make fine adjustments. On the other hand, during treatment, fiducial markers are used as indicators for marker setup, but there are cases where a deviation of nearly 10 mm is observed [28–31], and in this case, there is a risk that the pubic bones dose will increase more than planned. However, there have been no reports investigating how much internal error correction affects to increased pubic bones dose. In modern PT for prostate cancer, where extreme hypofractionation is progressing, it is considered important to conduct preliminary research on unknown issues. The purpose of this study was to investigate the tendency of pubic bones dose increase based on simulated internal error corrections on prostate PT with PS method.

## MATERIALS AND METHODS

### Structure delineation and treatment planning

Thirty consecutive patients with clinically localized prostate cancer who underwent PSPT at our institution were enrolled in this study. Our institutional review board approved this study (Approval number; 602). The median age was 73 years (range: 55–87 years). Melthea (Hitachi, Kashiwa, Japan) was used for the PT machine. The patients were placed in a supine position, and the lower legs were fixed with a vacuum cushion to reproduce the femoral heads. The fiducial markers (Gold Anchor, Naslund Medical Aktiebolag, Huddinge, Sweden) were implanted in the prostate to irradiate the target accurately, and a hydrogel spacer (SpaceOAR, Boston Scientific, Marlborough, United States of America) was inserted between the prostate and rectum to reduce the incidence of rectal bleeding. Thirty to sixty minutes prior to the treatment planning computed tomography (CT) scan, the patients were instructed to drink 200 ml water to ensure that the bladder was comfortably full. The patients were also instructed to defecate and exhaust gas as much as possible before the examination. An Aquilion LB (Canon Medical Systems, Otawara, Japan) was used for the CT scans, and images were taken in 2-mm slices. The CT images were transferred to the XiO-M treatment planning system (Hitachi, Kashiwa, Japan), where the clinical target volume (CTV) and OARs, such as the rectum, bladder and pubic bones were delineated by a single physician to minimize potential bias associated with multiple planners when comparing treatment plans. The pubic bones were delineated with the most cranial slice meeting the acetabulum and inferiorly to a horizontal line through the obturator foramen as reported by Kronborg *et al*. [24]. The CTV consisted of the prostate gland plus the proximal seminal vesicles. The planning target volume (PTV) included the CTV plus a 7-mm safety margin, except at the prostate gland-rectum interface, where a 6-mm margin was used to decrease the risk of rectal toxicity.

**Fig. 1:**
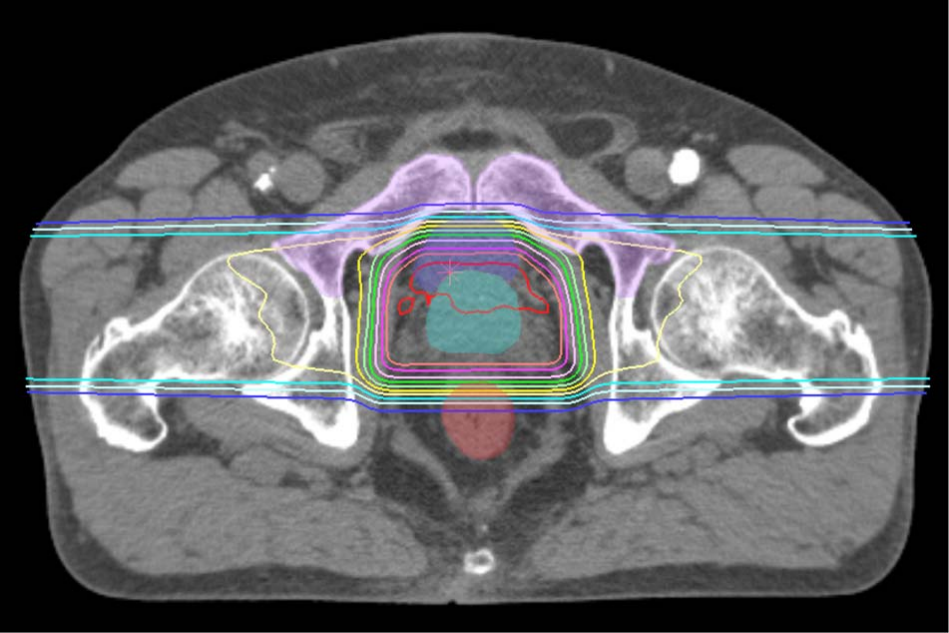
A typical dose distribution of passive scattering proton therapy using lateral opposed fields for prostate cancer. The clinical target volume, rectum, bladder, and pubic bones are contoured. The 100%, 95%, 90%, 80%, 70%, 60%, 50, 40%, 30%, 20% and 10% isodose lines are denoted in sequential order.

The irradiation method is the wobbler method, which is one of the PS methods [32]. The PSPT plans were designed using the standard lateral opposed fields with 210 MeV proton beams. The key parameters for the PSPT plans are distal, proximal, lateral and smearing margins. Most of the planning parameters are selected using the methods described by Moyers *et al*. [33]. The compensator was designed for the CTV using custom distal margin that included a 3.5% of depth to account for uncertainty for CT number accuracy and conversion to proton relative linear stopping power and a 3-mm range uncertainty to consider uncertainties in the accelerator energy, variable scattering system thickness and compensator density. The radiation field was formed using the multi-leaf collimator built in the snout. The prescribed dose was set to 63 Gy relative biological effectiveness (RBE) in 21 fractions to 95% of the PTV. Here, the RBE correction factor for physical to biological dose was 1.1. Dose constraints for OARs were in accordance with our institutional protocol, but adjustments were made to prevent 90% of the prescribed dose from reaching the circumference of the pubic bones. Figure  1 shows a typical dose distribution at our institution.

### Analysis

#### Analysis of pubic bones dose change due to internal error correction

It is known that interfractional internal error in the prostate can reach as much as 10 mm [28–31], and our experience has confirmed a similar trend. When the prostate moves anteriorly or inferiorly, the radiation field moves closer to the pubic bones, which is thought to increase the pubic bones dose. Therefore, we created a group of plans in which the isocenter of the reference plan was shifted assuming that the prostate shifted anteriorly or inferiorly by a maximum of 10 mm and in 2-mm increments, respectively. The irradiation conditions were all the same as in the reference plan before the shift. The increase in pubic bones dose in the shifted plan compared to the reference plan was analyzed using dose-volume histogram. Ideally, it would be desirable to use a more clinically meaningful indicator, such as a normal tissue complication probability model for pubic bone fractures, but because the tolerance dose with pubic bone fractures as the endpoint has not been clarified, in this study, the high dose ranges of V_80%_, V_90%_ and V_95%_ were used as the evaluation dose indices. Kronborg *et al*. reported that there was a borderline significant difference between the fracture and non-fracture groups in the volume of the pubic bones exposed to 40 Gy or more [24], but in this study, the high-dose range, which is thought to be at higher risk, was used as the analysis dose index. As can be seen from Fig.  1, dose concentration is high at 50% dose or more, and it is expected that the trends for V_50%_ and V_80%_ will be comparable. Therefore, it was deemed appropriate to use V_80%_, V_90%_ and V_95%_ as analysis dose indexes. Here, V_n%_ represents the volume at which n% of the prescribed dose of each OAR is irradiated. The 50%, 80%, 90% and 95% doses correspond to 33.0 Gy(RBE), 52.8 Gy(RBE), 59.4 Gy(RBE) and 62.7 Gy(RBE), respectively, which are converted to 29.7 Gy(RBE), 57.0 Gy(RBE), 67.7 Gy(RBE) and 73.4 Gy(RBE) in equivalent doses in 2 Gy(RBE) fractions (assuming α/β = 3).

#### Correlation between pubic bones dose change and shortest distance between CTV and pubic bones

It is expected that the effect of internal error correction on the pubic bones may depend on the anatomical distance between the CTV and the pubic bones. Therefore, we analyzed the Pearson’s correlation between the shortest distance between the CTV and the pubic bones and pubic bones dose, as well as the between the shortest distance between the CTV and the pubic bones and increasing trend of pubic bones dose when the isocenter was shifted. Using the automatic margin function of the treatment planning system, the shortest distance was defined as the value at which the margin added to the CTV touched the pubic bones. All statistical analyses were performed using SPSS version 29.0.2.0 (IBA, Armonk, NY, USA).

## RESULTS

**Fig. 2:**
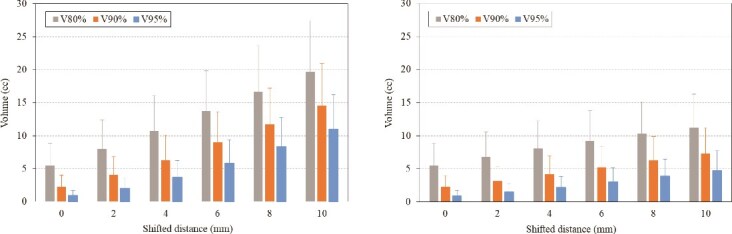
The results of V_80%_, V_90%_ and V_95%_ of pubic bones when the isocenter was shifted anteriorly (left) and inferiorly (right). The error bars indicate one standard deviation.

**Fig. 3:**
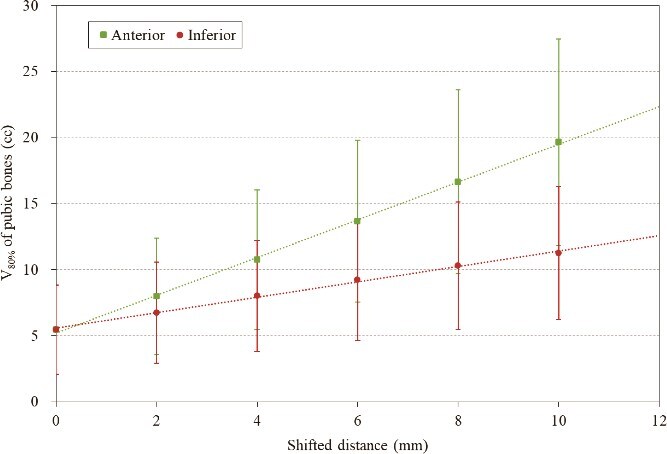
The trends of V_80%_ of pubic bones when the isocenter was shifted anteriorly and inferiorly. The error bars indicate one standard deviation.

**Fig. 4:**
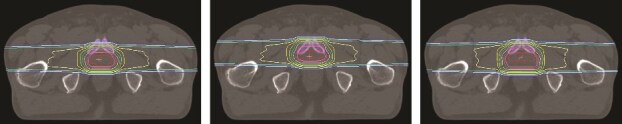
Dose distributions at the pubic bones slice level in a typical case with a reference plan (left), 10-mm anterior shifted plan (center) and 10-mm inferior shifted plan (right). The pubic bones are contoured. The 100%, 95%, 90%, 80%, 70%, 60%, 50, 40%, 30%, 20% and 10% isodose lines are denoted in sequential order.

The results of V_80%_, V_90%_ and V_95%_ when the isocenter was shifted anteriorly and inferiorly are shown in Fig.  2. The distance between the CTV and the pubic bones differed depending on the patient, and in cases where there was a large distance, V_95%_ was almost zero even after shifting. For all dose indices, the increase in pubic bones dose tended to be greater when shifting anteriorly than inferiorly. As shown in Fig.  3, both anterior and inferior shifting V_80%_ showed a nearly linear increase with shift distance. However, the slope of the increase was visibly steeper in the anterior shifting than in the inferior shifting, indicating a marked difference in the rate of change between the two shift directions. Compared to the reference plan, the 10-mm shifted plan resulted in an increase of 14.2 cc and 5.8 cc in V_80%_ on average anteriorly and inferiorly, respectively, with the anterior increase being ⁓2.4 times greater. Figure  4 shows the dose distribution at the pubic bones slice level in a typical case with a 10-mm anterior and inferior shift. It can be seen that in some areas, ˃90% of the dose is irradiated circumferentially around the pubic bones.

**Fig. 5:**
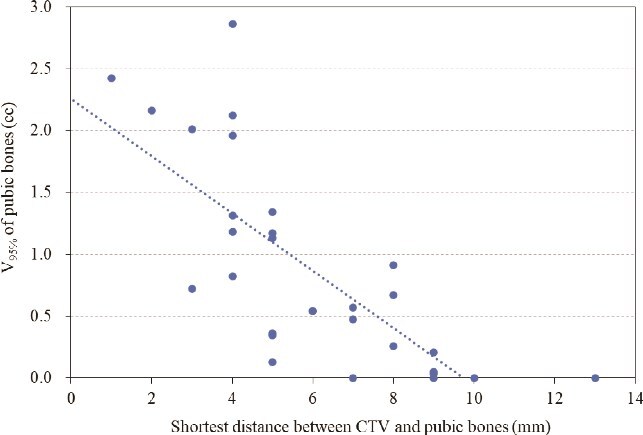
Scatter plots with regression line describing relationship between the shortest distance between the CTV and the pubic bones and V_95%_ of pubic bones for the reference plan.

**Fig. 6:**
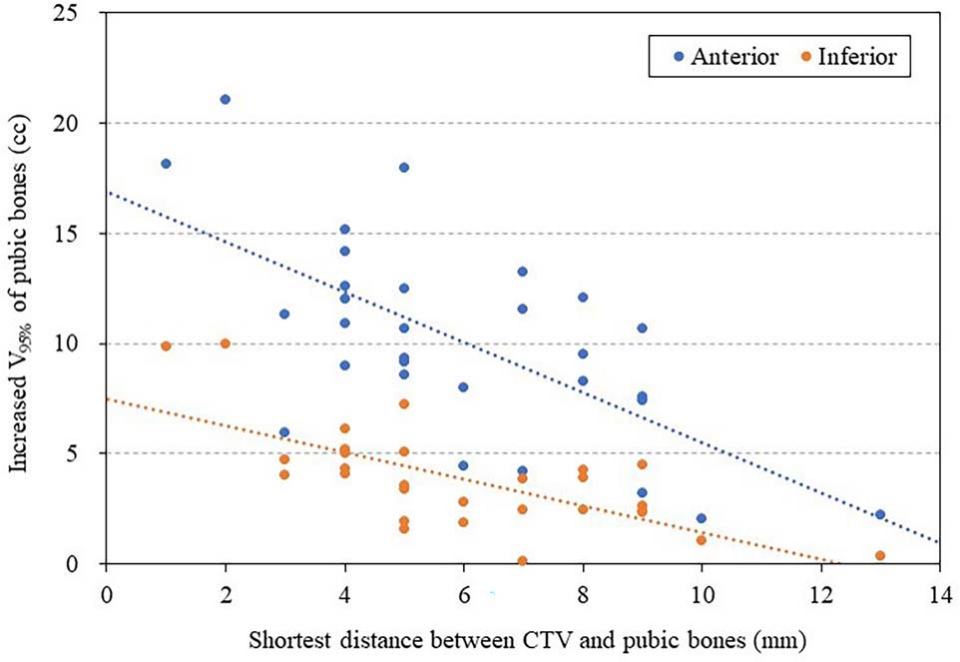
Scatter plots with regression lines describing relationships between the shortest distance between the CTV and the pubic bones and increased V_95%_ of pubic bones for the 10-mm anterior shifted plan and 10-mm inferior shifted plan.

Figure  5 shows the correlation between the shortest distance between the CTV and the pubic bones and V_95%_ of pubic bones for the reference plan. A significant correlation was found between the shortest distance between the CTV and the pubic bones and V_95%_ of pubic bones for the reference plan (*r* = 0.749, *P* < 0.001), with similar trends observed for V_80%_ and V_90%_. Figure  6 shows the correlation between the shortest distance between the CTV and the pubic bones and increased V_95%_ of pubic bones with a 10-mm anterior and inferior shift. A significant correlation was found between the shortest distance between the CTV and the pubic bones and increased V_95%_ of pubic bones in a 10-mm anterior shift (*r* = 0.662, *P* < 0.001) and in a 10-mm inferior shift (*r* = 0.695, *P* < 0.001). The same trend was seen in the V_80%_ and V_90%_.

## DISCUSSION

The site of the pelvic bone that may be irradiated in external radiation therapy for prostate cancer is the pubic bones, and it has been shown that pubic bone fractures can actually occur [16]. Although this report differs from the high-precision radiation therapy techniques currently in use, it is noteworthy that it highlights the risk of fractures when relatively high doses are delivered to the pubic bones. While such fractures have not been observed at our institution, we were informed through personal communication that there have been cases of pubic bone fractures in PSPT for prostate cancer. It has also been pointed out that this may cause chronic lower extremity pain and radiation-induced neuropathy [34]. Although there are few reports on these symptoms, we recognize them as known reactions based on personal communication. Therefore, this is an important factor to be considered during treatment planning. Although personal communication does not constitute scientific evidence, the current inability to provide clear answers to issues frequently encountered in daily clinical practice in PSPT should be addressed. Clarifying this matter is considered to have substantial significance. In particular, since it is expected that internal error correction will have the greatest effect on pubic bones dose in PSPT, we attempted to examine this in this study. As a result, although there were slight differences in trends for each case, it was found that, overall, there was a tendency for the pubic bones dose to increase particularly when shifting anteriorly compared to the inferiorly. It was also found that the smaller the shortest distance between CTV and pubic bones, the greater the impact on the pubic bones dose during internal error correction. Although several studies have suggested that the dose–response relationship may be influenced by factors such as the high-dose regions, V30–50Gy, or fraction dose, a definitive dosimetric parameter has yet to be established [23, 24, 35]. Kronborg *et al*. reported that there was a borderline significant difference between the fracture and non-fracture groups in the volume of the pubic bones exposed to 40 Gy or more [24]. As shown in Fig.  1, dose concentration remains high up to the 50% isodose level, suggesting that similar trends may persist at higher dose levels. Furthermore, as illustrated in Fig.  3, the increase in pubic bones dose appears to exhibit an approximately linear relationship with the magnitude of positional shift within the 50–95% dose range. These findings indicate that anterior shift could, relative to inferior shift, increase the risk of fracture. On the other hand, Rasmusson *et al*. conducted a long-term observation of pelvic fractures in 346 cases of external radiation therapy for prostate cancer [15]. They reported that the mean D_2%_ (near-maximum dose) to the pubic bones was 77.0 Gy, but there was no significant increase in the incidence of pubic bone fractures compared to the control group that did not receive irradiation. However, pubic bone fractures were still observed in 2% of cases, and this finding cannot be interpreted as conclusive evidence that high-dose radiation to the pubic bones has no clinical consequences. Given the current lack of solid scientific evidence, we believe it is prudent to interpret the findings with caution without excluding potential risks.

In practice, a 10-mm displacement is rare, but our results suggest that early replanning should be considered if the displacement persists for any reason, especially in the anterior direction. Simulation studies often model extreme conditions beyond realistic error limits to elucidate their potential impacts, thereby enhancing our understanding of underlying mechanisms. For instance, Yoon *et al*. evaluated the effects on dose distribution of target displacements up to 15 mm in each direction [36]. Although a 15 mm lateral displacement of the prostate is highly improbable in clinical practice, such simulations can yield valuable insights. Notably, their analysis addressed the CTV, rectum and bladder but did not consider bony structures, such as the pubic bones. In our study, a maximum displacement of 10 mm—representing the upper bound of clinically plausible motion—was assumed, which should assist readers in interpreting our findings. Since prostate cancer cases are often undergoing hormone therapy and also have advanced osteoporosis, the risk may be higher than that of healthy people. It has been noted that the effects vary depending on the irradiation technique [18, 23], therefore, during treatment planning, it is advisable to check the dose to the pubic bones in addition to the dose to the rectum and bladder. It goes without saying that genitourinary and gastrointestinal toxicities must be given priority from a clinical perspective. However, in extreme hypofractionated protocols in particular, it is desirable to consider the effects on surrounding organs such as the pubic bones in order to maintain the patient’s quality of life. In addition, when IGRT is used in combination, it is advisable to assume that internal error correction will be performed and to discuss the effects on surrounding normal tissues, including the pubic bones. It is also important for medical staff to share information so that if unexpected prostate displacement occurs during treatment, they can discuss whether or not to continue treatment.

This study has some limitations. First, this study only included cases in which hydrogel spacer, which has become widely used in recent years [7], was used. The presence or absence of hydrogel spacer may result in changes in the anatomical distance between the prostate and the pubic bones. Further verification is required to determine whether similar results can be obtained without using hydrogel spacer. Second, the analysis was conducted only on the PS method performed at our institution. The method of forming the irradiation field in PT varies from system to system, even if the same PS method is used, so the tendency may be different with different systems. In addition, PBS has not been considered, and this is thought to be an issue that needs to be addressed in the future. In this study, the treatment plan was analyzed assuming lateral opposed fields, one on the left and one on the right, would be used per day, but there are quite a few facilities that use a method in which one field is used per day, changing the direction each day. In particular, with hypofractionated irradiation, if the internal error correction becomes large, the risk of pubic bone fracture may increase more than expected, so sufficient caution is required.

## CONCLUSION

The effect of internal error correction on pubic bones dose during PSPT for prostate cancer using lateral opposed fields was analyzed. It was found that the pubic bones dose tended to increase more when the isocenter was shifted anteriorly than inferiorly. When performing internal correction using IGRT, there is a risk of an unexpectedly high dose being irradiated to the pubic bones, especially if a relatively large systematic misalignment continues anteriorly. As hypofractionation progresses, the risk is expected to increase, so in such cases, it is desirable to take measures such as considering early replanning.

## Presentation at a conference

None.

## Clinical trial registration number

None.
